# A case of splenic rupture a week after appendectomy

**DOI:** 10.1093/jscr/rjab541

**Published:** 2021-12-11

**Authors:** Catherine Deleuze, Celine Rasmont, Todor Ivanov, Nicolas Brassart, Malek Ghaddab, Laura Romero Stoca, Didier Hossey, Tsy-Yeng Choy, Jean Lemaitre

**Affiliations:** General Surgery Department, Ambroise Paré Hospital, Mons, Belgium; General Surgery Department, Ambroise Paré Hospital, Mons, Belgium; General Surgery Department, Ambroise Paré Hospital, Mons, Belgium; Radiology Department, Ambroise Paré Hospital, Mons, Belgium; Radiology Department, Ambroise Paré Hospital, Mons, Belgium; General Surgery Department, Ambroise Paré Hospital, Mons, Belgium; General Surgery Department, Ambroise Paré Hospital, Mons, Belgium; General Surgery Department, Ambroise Paré Hospital, Mons, Belgium; General Surgery Department, Ambroise Paré Hospital, Mons, Belgium

**Keywords:** Atraumatic splenic rupture, Acute appendicitis, E.Coli bacteriemia

## Abstract

A 52-year-old woman developed atraumatic splenic rupture 1 week after appendectomy for perforated appendicitis. The emergency computed tomography (CT) revealed abscessed appendicitis. We performed a laparoscopic appendectomy and meticulous peritoneal lavage of the right lower quadrant peritonitis. Intravenous antibiotics were prolonged after surgery. Six days after appendectomy, she presented acute signs of hypotensive shock associated with abdominal pain and blood in the pelvic drain. Emergency CT scan revealed splenic rupture with major hemoperitoneum and active splenic bleeding. Embolization of the splenic artery was initially successful, but she relapsed into shock a few hours later. We proceeded to splenectomy. Pathological examination only found inflammation. She was discharged 1 month after the initial operation. Spontaneous splenic rupture is a rare but life-threatening complication of appendicitis with major peritonitis. It must be identified and treated immediately. Colic microbiota could be responsible of acute splenitis and congestion after a bacteremia.

## INTRODUCTION

We describe the case of a 52-year- old woman who developed an atraumatic splenic rupture (ASR) 1 week after appendectomy for perforated appendicitis. The emergency computed tomography (CT) revealed inflammation in lower right abdomen and small amount of ascites in pelvis. Histopathological examination found no abnormalities except inflammation.

ASR is a rare surgical emergency, which is often associated to neoplastic (30%) or infectious (27%) causes. The infectious disorders known in these situations are often *Pneumococcus, Meningococcus, Cytomegalovirus* and *Plasmodium falciparum* [1].

We found only five other case reports in literature related to another abdominal infection origin.

## CASE REPORT

A 52-year-old woman presented at the emergency department with pyrexia (38,3°C), lower abdominal pain and tenderness in the right iliac fossa. The pain lasted since the night before. She complained of vomiting.

The blood tests showed elevated inflammatory parameters: leucocytes: 19.4 × 10^9^/l (91% of Neutrophils) and C-reactive protein: 312 mg/l.

**Figure 1:**
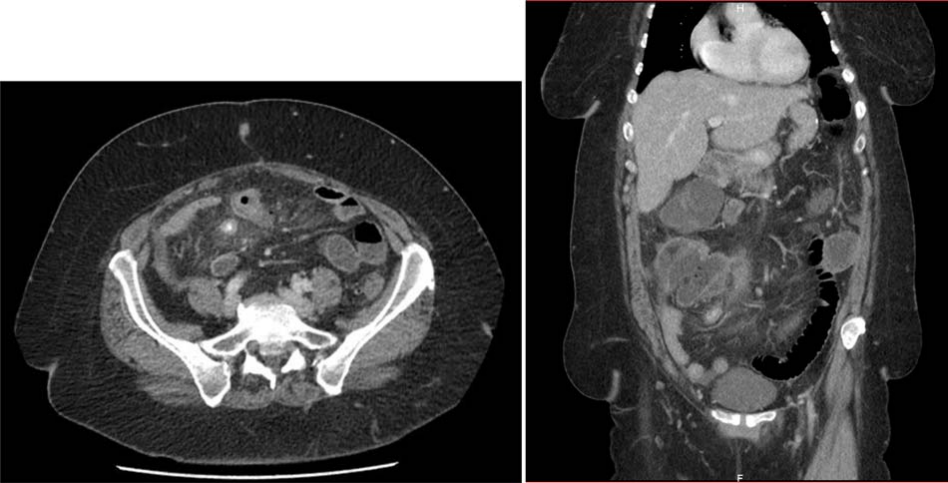
Emergency CT: acute appendicitis with: intra-appendicular liquid, microstercoliths and peri-appendicular inflammation.

Abdominal CT showed signs of non-complicated appendicitis: intra-appendicular liquid, microstercoliths and peri-appendicular inflammation (Fig. 1).

The patient underwent appendectomy for acute appendicitis progressing in <24 hours.

The main operative finding was a purulent peritonitis of the right abdomen. There was a lot of inflammation into the ileocecal mesentery. We performed a laparoscopic appendectomy and meticulous peritoneal lavage. We left in place two abdominal drains (Jackson Pratt).

Because of purulent peritonitis and presence of *Escherichia Coli* in blood cultures, intravenous antibiotherapy (amoxicillin and clavulanic acid) was prolonged after surgery for 5 days.

**Figure 2:**
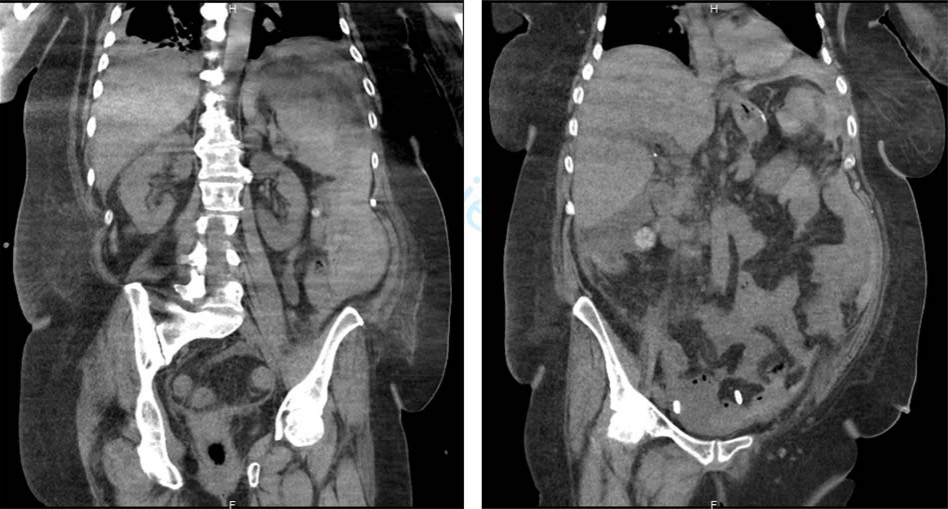
Splenic subcapsular hematoma and major hemoperitoneum.

On the sixth post-operative day, the patient suddenly felt weak. She presents with pallor, hypotension and tachycardia. We noticed some blood in the left drain. We immediately started a fluid resuscitation and performed an urgent CT which showed a fresh 6-cm wide splenic sub capsular hematoma associated with a major hemoperitoneum (Fig. 2).

**Figure 3:**
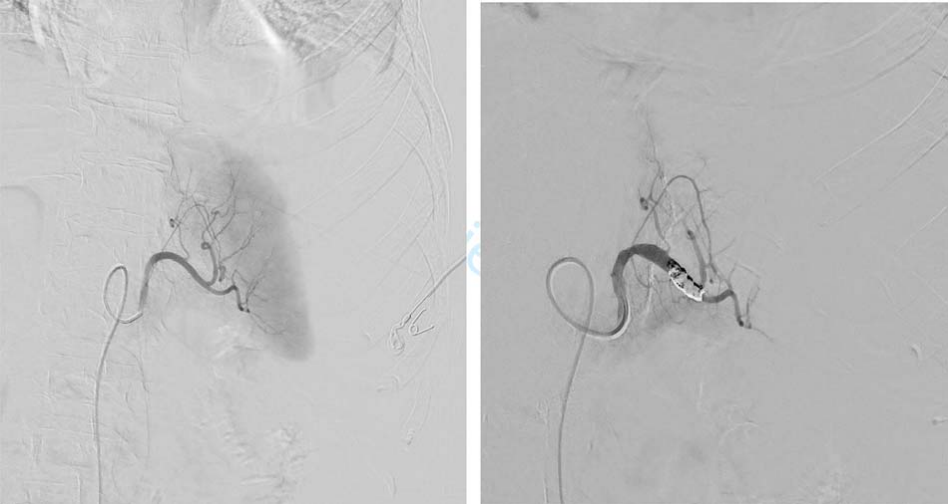
Embolization of splenic artery.

First, we embolized the splenic artery with coils (Fig. 3). The patient was getting well at intensive unit care, but few hours after embolization, she was shocked again.

We decided then to perform an emergency laparotomy that revealed a massive splenic hematoma and a totally burst spleen. We carried out the splenectomy and controlled the splenic vessels.

A paralytic ileus and a secondary pleural effusion marked the second post-operative period. The patient has recovered in 2 weeks and was finally discharged 1 month after initial admission for acute appendicitis.

## DISCUSSION

ASR is a rare but life-threatening complication of acute appendicitis.

A systematic review of 63 publications and 845 patients estimates the ASR mortality rate at 12% irrespective of etiology [1, 2].

ASR is often associated to neoplastic (30%), infectious (27%) or inflammatory causes (20%). The infectious disorders known in these situations are often Pneumococcus, Meningococcus, Cytomegalovirus and *P. falciparum* [1, 2].

In our case, the blood cultures showed an *E. Coli* bacteremia. This could explain the rupture mechanism: the patient developed a bacteremia with septic embolization of the spleen. This led to splenic hypertension and rupture [3, 4].

Full virological studies confirm the absence of recent viral infection [4].

Despite the 845 ASR review, we found only two similar cases [3, 5]. Our patient is the only one with *E. Coli* infection.

The histopathological examination found inflammation in the splenic parenchyma.

The nomenclature of ASR is quite vague. Authors use many different terms to discuss about same entities. P. Renzulli *et al*. proposed a simplified classification of splenic rupture [1]. This classification is described in Table 1.

**Table 1:** Classification of splenic rupture by Renzulli *et al*. [1]

Traumatic rupture	With adequate trauma	
Atraumatic rupture	Without adequate trauma	
	Atraumatic pathological splenic rupture (93%)	With histopathological changes
	Atraumatic idiopathic splenic rupture (7%)	Without hispathological changes

As for traumatic splenic rupture, the hemodynamic stability determines the choice of the treatment. A non-operative management is acceptable if the patient is stable.

Because our patient improved her status with fluid resuscitation, we decided to embolize first to avoid surgery. Unfortunately, she was back in shock few hours later and we had to proceed to splenectomy.

In ASR, it makes sense to perform splenectomy even if the patient is stable because of the histopathological examination which will establish the etiology [1].

## CONCLUSION

ASR is a rare but life-threatening complication of acute appendicitis. It must be recognized as soon as possible and should be treated immediately by embolization or splenectomy.

The mechanism could be bacteremia and major splenic inflammatory response which generate splenic hypertension and rupture.

## CONFLICT OF INTEREST STATEMENT

The authors have no conflict of interest to declare.

## FUNDING

No funding received.
